# C3 glomerulopathy

**DOI:** 10.12688/f1000research.10364.1

**Published:** 2017-03-10

**Authors:** H. Terence Cook

**Affiliations:** 1 Department of Medicine, Imperial College London, Hammersmith, London, UK

**Keywords:** C3 complement, glomerular inflammation, renal pathology

## Abstract

C3 glomerulopathy is a recently defined entity that encompasses a group of kidney diseases caused by abnormal control of complement activation with deposition of complement component C3 in glomeruli leading to variable glomerular inflammation. Before the recognition of the unique pathogenesis of these cases, they were variably classified according to their morphological features. C3 glomerulopathy accounts for roughly 1% of all renal biopsies. Clear definition of this entity has allowed a better understanding of its pathogenesis and clinical course and is likely to lead to the design of rational therapies over the next few years.

## Introduction

Classification of kidney pathology has traditionally relied on separating diseases into categories depending on their light microscopic appearances. However, it is much more useful to classify on the basis of pathogenesis. An excellent example of the change from morphology-based to pathogenesis-based classification is the recent description of the entity of C3 glomerulopathy ^1^ caused by uncontrolled activation of the alternative pathway of complement. In this review, I will describe the complement system and the ways in which defects in control can lead to C3 glomerulopathy. I will outline what is known of the pathological and clinical features and describe the outstanding questions in this disease.

## The complement system and the glomerulus

The complement system comprises over 30 proteins in circulation or on cell membranes. It has a central role in defence against micro-organisms and in clearance of apoptotic cells and debris. The complement cascade may be activated in several ways but central to all of them is the formation of an enzyme that cleaves C3, generating fragments C3a and C3b. Rapid amplification of the pathway is then achieved through a feedback loop that generates more C3b. The classic pathway is activated by antigen-antibody complexes and proceeds via C1, C2 and C4. The lectin pathway is activated by carbohydrate groups on micro-organisms and also involves cleavage of C2 and C4. The alternative pathway, which is the most primitive in evolutionary terms, is unique in that it is continually active in the circulation as a consequence of the spontaneous hydrolysis of C3, allowing the formation of a C3 convertase. This ensures that the system is ready to respond rapidly to foreign surfaces such as micro-organisms. Because of this spontaneous activity of the alternative pathway and the rapid amplification loop, the activity of the pathway needs to be tightly controlled. The major inhibitor of the alternative pathway in the circulation is factor H which acts to block the formation of alternative pathway convertases, promotes their spontaneous dissociation and also acts as a co-factor for the cleavage of C3b to its inactive form iC3b by factor I. Factor H is composed of 20 protein subunits (each approximately 60 amino acids), known as short consensus repeat (SCR) domains. The complement-inhibiting activity of factor H resides within the first four N-terminal SCRs. The two C-terminal SCRs (SCR 19 and 20) are responsible for the ability of factor H to bind to self cell surfaces such as endothelium and locally inhibit the alternative pathway. The complement activation cascade and the role of factor H and its related proteins were recently reviewed ^2^.

The importance of factor H in inhibiting the alternative pathway is demonstrated by mice with a targeted deletion of factor H ^3^. These mice have uncontrolled activation of the alternative pathway in the circulation and thus have very low circulating levels of C3. From 4 days of age (the earliest time point examined), they have deposition of C3 on the glomerular basement membrane with subsequent development of electron-dense deposits seen on electron microscopy (EM) by 2 months of age. This leads to glomerular inflammation and structural changes with the pattern of a membranoproliferative glomerulonephritis (MPGN)—that is, glomerular architectural changes characterised by mesangial expansion and hypercellularity and by thickening of the glomerular capillary wall ^3^. The pathological significance of inhibition of the alternative pathway in the fluid phase compared with inhibition on cell surfaces was elegantly demonstrated by taking the factor H-deficient mice and making them transgenic for a form of factor H lacking the last five SCRs of factor H ^4^. These mice were able to regulate the alternative pathway in the circulation and had normal levels of C3 but were unable to control activation on the endothelium, leading to a renal thrombotic microangiopathy as seen in human atypical haemolytic uremic syndrome.

In humans, the presence of isolated C3 deposits in glomeruli, detectable by immunofluorescence and seen as deposits on EM, which are due to abnormal control of complement activation, has been given the name of C3 glomerulopathy ^1^. Before the recognition of this as a distinct pathological process, most of these cases would have been classified on the basis of their morphological appearance and many of them would have been labelled as MPGN. It is important to be aware that C3 in renal biopsies is usually detected with an antibody to C3c which reflects recent C3 activation ^5^.

## Pathology of C3 glomerulopathy

**Figure 1.**
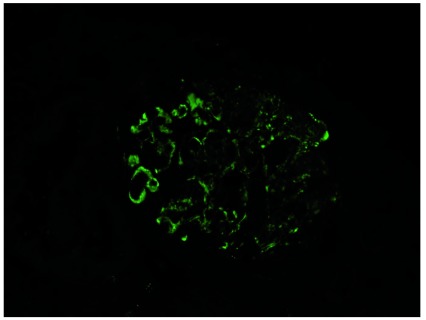
C3 staining in C3 glomerulopathy. Glomerulus showing staining for C3 in a case of C3 glomerulopathy. The kidney is stained with a fluorescently labelled antibody to C3.

Glomerulonephritis due solely to alternative pathway activation would be expected to show C3 in glomeruli on immunofluorescence with no immunoglobulins, C1q or C4 ( Figure 1). This is the finding in many cases, but (as discussed below) some cases that are almost certainly due to defects in alternative pathway activation may have small amounts of immunoglobulin, possibly because they are triggered by immune complex deposition. With stringent criteria requiring the presence of C3 and electron-dense deposits but no IgA or IgG deposition, C3 glomerulopathy was found in 1.34% of biopsies in a single centre with an estimated incidence of 2 per million population per year ^6^.

**Figure 2.**
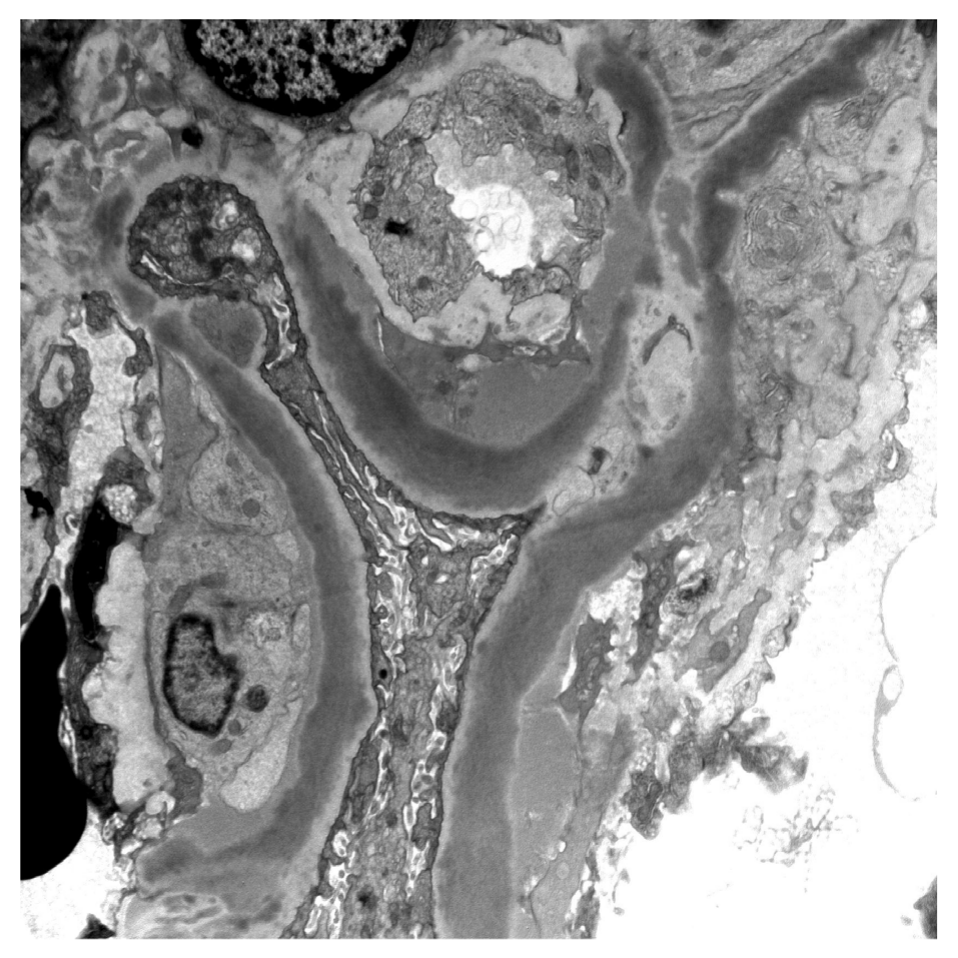
Electron micrograph showing dense transformation of the glomerular basement membrane in a case of dense deposit disease.

**Figure 3.**
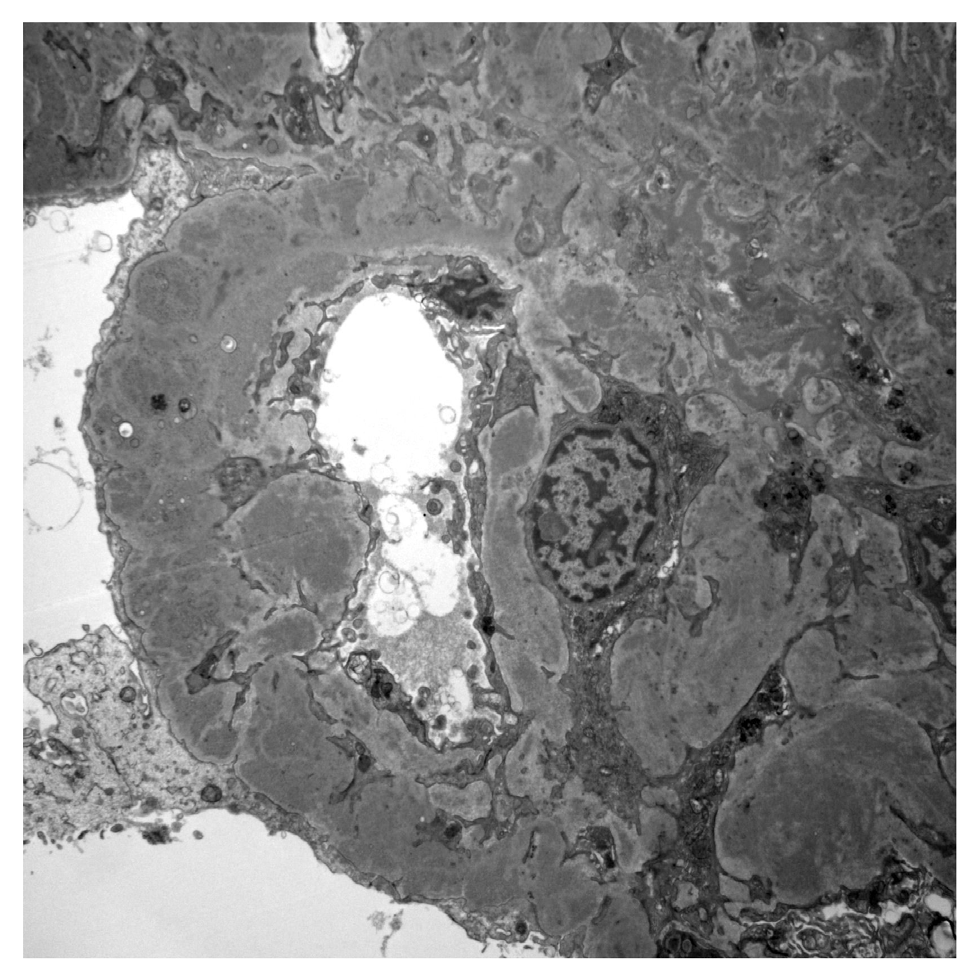
Electron micrograph showing multiple electron-dense deposits in the mesangium and capillary wall of a glomerulus in a case of C3 glomerulonephritis.

Cases of C3 glomerulopathy show variable appearances on light microscopy and EM. On EM, some biopsies show dense osmiophilic transformation of the glomerular basement membrane with similar dense deposits in the glomerular mesangium and sometimes on Bowman’s capsule and in tubular basement membranes. This is referred to as a dense deposit disease (DDD) ( Figure 2) and has been recognised as a distinct morphological appearance for over 50 years ^7^. In most cases, the deposits do not have this dense appearance but may form ill-defined electron densities within the basement membrane or mesangium or may resemble the deposits typically seen in immune complex glomerulonephritis with deposits in sub-endothelial or sub-epithelial locations ( Figure 3); this group is by convention referred to as C3 glomerulonephritis (C3GN). It is important to understand that, in this potentially confusing terminology, C3 glomerulonephritis is thus a subset of C3 glomerulopathy that does not show the morphological features of DDD. It is not known why, in some cases, the deposits of C3 lead to the typical appearances of DDD. It is important to realise that the assessment of the density of the deposits is subjective and there will be some cases where pathologists would disagree about whether to classify them as DDD or C3GN ^8, 9^. In large series, the ratio of cases of C3 glomerulopathy that have DDD morphology to C3GN is approximately 1:3 ^6, 10^. There are no clear-cut clinical distinctions between DDD and C3GN, although patients with DDD are more likely to have low levels of C3 in circulation ^6^ and to have a C3 nephritic factor ^10^, an autoantibody that stabilises the alternative pathway C3 convertase.

In general, with careful attention to immunohistology and EM, the distinction of C3 glomerulopathy from thrombotic microangiopathy is straightforward. However, difficulties may arise if EM is not available since cases of chronic thrombotic microangiopathy may have double contours of glomerular capillary walls on light microscopy. However, in general, they do not show strong capillary wall staining for C3c. In rare cases, the features of a coexistent C3 glomerulopathy and thrombotic microangiopathy may be seen in the same biopsy (personal observation).

**Figure 4.**
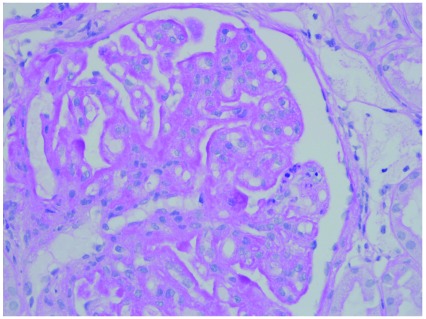
Glomerulus from a case of C3 glomerulonephritis showing a membranoproliferative glomerulonephritis pattern. There is increased mesangial matrix and mesangial cells. Capillary walls are thickened with segmental double contours.

By light microscopy, as noted above, many cases of C3 glomerulopathy, whether DDD or C3GN, show an MPGN morphology ( Figure 4). In one series, 71% of C3GN cases had an MPGN pattern ^10^. In most of the others, the pattern will be of mesangial proliferation without capillary wall changes. On this background pattern of disease, glomeruli may show variable inflammatory cell infiltration, crescent formation and glomerular sclerosis ^8, 11, 12^. Clinical presentation is variable but the majority of patients have a slowly progressive course with a 10-year renal survival of approximately 50% ^10^. Some patients present with a rapidly progressive crescentic glomerulonephritis ^6, 10, 12– 15^.

In some patients, activation of C3 leads to subsequent generation of a C5 convertase and generation of the terminal complement complex C5b-9. This may be detected in glomeruli in biopsies ^16^ and in circulation ^17^. It has been suggested that those patients with circulating C5b-9 have activation of the alternative pathway in the plasma but that those without have activation on glomerular surfaces.

C3 glomerulopathy recurs in transplant kidneys. DDD is reported to recur in virtually all allografts and 50% are lost due to recurrent disease ^18^. As C3GN has been recognised only recently, the rate of recurrence is not as well defined, but there are several reports of recurrence ^16, 19, 20^.

## Difficulties in diagnosis

The initial concept of C3 glomerulopathy was of glomerular injury characterised by immunostaining of glomeruli for C3 but with no immunoglobulin or C1q detected ^1^. However, it is clear that these criteria are too restrictive and that many cases that are due to failure to control alternative pathway activation may show some immunoglobulin in glomeruli ^21^. It is possible that this is because low-grade immune complex deposition triggers the disease or its clinical presentation. Evidence suggests that, on immunofluorescence, the criterion with the best balance of sensitivity and specificity is the presence of dominant C3 staining with the intensity of C3 staining at least two orders of magnitude ^22^ greater than any other immunoreactant (that is, IgG, IgM, IgA and C1q) ^21, 23^. In some cases, the initial kidney biopsy may not show C3-dominant GN but subsequent biopsies may ^21, 24, 25^, suggesting that in cases with an atypical clinical course repeat biopsies may be useful. However, these problems in diagnosis draw attention to the fact that the distinction between C3 glomerulopathy and immunoglobulin-associated MPGN is not always clear-cut. It has been suggested that the presence of C4d staining might help to distinguish immunoglobulin-associated GN ^22^, but a recent consensus report concluded that further studies were necessary to validate this ^23^.

A potential pitfall in diagnosis is the occasional existence of what have been called ‘masked monoclonal immunoglobulin deposits’, which are glomerular deposits of monoclonal immunoglobulin that cannot be detected by standard immunofluorescence of frozen sections but are detected by immunofluorescence performed on protease-digested paraffin sections ^26, 27^. In one series, the authors described 10 patients with MPGN that would have been diagnosed with C3 glomerulopathy if the presence of monoclonal immunoglobulin had not been revealed in paraffin sections ^27^.

Post-infectious glomerulonephritis is the name given to a self-limiting glomerulonephritis that most commonly develops as a consequence of streptococcal infection. Renal biopsy typically shows a diffuse endocapillary glomerulonephritis with sub-epithelial hump-like deposits on EM. On immunofluorescence, there may be glomerular staining for IgG and C3 but some cases may show C3 deposition only ^28^. Patients usually have low levels of circulating C3. Therefore, post-infectious glomerulonephritis could be considered a self-limiting form of C3 glomerulopathy. Many cases have now been reported in which the initial presentation appears to be post-infectious GN but the patients have gone on to develop a chronic C3 glomerulopathy ^29– 31^. Therefore, in any patient with a C3-dominant glomerulonephritis with features of post-infectious GN, persistent clinical abnormalities, including hypocomplementaemia, proteinuria or declining renal function, should lead to further investigation of the alternative pathway of complement ^8, 28^.

## Pathophysiology

There are many mechanisms that can interfere with inhibition of the alternative pathway. I shall discuss genetic causes first and then acquired causes. Many genetic variants have been associated with C3 glomerulopathy. In some cases, these are rare variants that are associated with familial disease providing strong evidence that they are causative. Others are rare variants found in individual patients and thought likely to be pathogenic, and finally there are common polymorphisms that increase the risk of disease in association with other genetic variants or environmental factors. In the first category, there are rare families reported with genetic deficiency of factor H ^32, 33^ or with a mutation in factor H that renders it less able to regulate the alternative pathway ^34, 35^. A family has been described with a variant of the C3 gene that coded for a version of C3 that formed a C3 convertase resistant to decay by factor H, and another pedigree showed a mutation of C3 leading to defective regulation by CR1 and factor H ^36^. Studies of familial C3 glomerulopathy have also revealed roles in complement activation for a group of factor H-related proteins (CFHRs). There are five human CFHRs that are coded downstream of factor H on chromosome 1. They have a high homology with factor H at their C-terminal ends. CFHRs 1, 2 and 5 are now known to dimerise and the dimers appear to be able to antagonise the activity of factor H on surfaces such as the glomerular basement membrane. Because of areas of high homology in this gene region, duplication and rearrangements are relatively common. Five separate abnormal FHR proteins have now been associated with C3 glomerulopathy ^37– 42^. In all of these cases, the abnormal proteins either show internal duplications or are fusion proteins that have duplication of the first two domains of the CFHRs. These are the domains involved in dimerisation and it is believed that the mutant proteins form oligomers that show enhanced antagonism of factor H. Interestingly, with three of these mutations, circulating C3 levels were reported as normal, implying that the dysregulation of the alternative pathway is within the glomerulus rather than in the circulation ^37, 39, 42^.

In addition to familial cases, other series have described sporadic rare genetic variants associated with C3 glomerulopathy. In a series of 73 patients with C3 glomerulopathy, likely pathogenic genetic variants were found in 18% of the patients; 14% had at least one relative with C3 glomerulopathy or renal impairment without another apparent cause ^17^. These included variants of genes coding for factor H, factor I, factor B, CD46, C3 and, in one patient, thrombomodulin. As well as rare pathogenic genetic variants, there are also common variants of complement genes that increase the risk of C3 glomerulopathy. These include variants of factor H and CD46 ^10, 17^. It is likely that in individual patients there is a multifactorial genetic contribution to C3 glomerulopathy ^43^.

The commonest acquired factors associated with C3 glomerulopathy are C3 nephritic factors (C3nefs), which are autoantibodies that bind to and stabilise the alternative pathway C3 convertase by preventing its inactivation by factor H ^44^. C3nefs can be identified in 40% to 60% of cases of C3GN and 80% to 90% of cases of DDD ^10, 17^. However, C3nefs are heterogeneous and there is a lack of standardised assays for their measurement, making comparisons between different cohorts difficult. In rare patients, autoantibodies to factor H, factor B or C3b have been identified ^45– 47^. There are several reports of an increased incidence of monoclonal gammopathy in patients with C3 glomerulopathy ^48– 50^. In a group of 14 patients with DDD over age 49, 10 had a monoclonal immunoglobulin ^49^, and in a group of 32 patients with C3GN, 31% had a monoclonal immunoglobulin with a mean age of 54.5 years ^50^. The mechanism by which monoclonal gammopathy predisposes to C3 glomerulopathy is not clear in most cases, but it has been suggested that the monoclonal immunoglobulin may act as an autoantibody to complement components. These findings emphasise the importance of testing for monoclonal immunoglobulin in older patients with C3 glomerulopathy.

A significant percentage of patients do not have a pathogenic genetic variant or a C3nef. Patients with a C3nef or a pathogenic genetic variant or both have lower levels of circulating C3 and higher levels of circulating C5b-9 than those without, suggesting that in these patients there is activation of the alternative pathway in the plasma ^17^. Interestingly, patients without a mutation or C3nef have a higher risk of end-stage renal disease ^17^. In two different cohorts ^10, 17^, the incidence of genetic variants or C3nefs in C3 glomerulopathy was very similar to the incidence in those in whom the disease was classified as immunoglobulin-associated MPGN, suggesting that the distinction between these entities is not clear-cut. As noted above, it is important to recognise that cases may be initially categorised as immune complex-MPGN but that the diagnosis of C3 glomerulopathy becomes apparent on subsequent biopsies ^24^.

Further insights into pathogenesis have come from animal models. Factor I is a circulating protein that acts together with factor H to cleave the active form of C3, C3b, to the inactive iC3b. Interestingly, if factor H-deficient mice are also deficient in factor I, they do not develop C3 deposition on the glomerular basement membrane but instead show mesangial C3 ^51^. This implies that it is iC3b that is the fragment of C3 that is deposited on the basement membrane. Properdin is the only positive regulator of the alternative pathway and acts to stabilise the alternative pathway C3 convertase. It might therefore be predicted that inhibition of properdin would protect from C3 glomerulopathy. Paradoxically, in the mouse model of factor H deficiency, lack of properdin exacerbated disease with increased glomerular C3 deposition ^52, 53^.

## Therapy

The optimal treatment for C3 glomerulopathy remains undefined. Most information relates to DDD since C3GN was not previously recognised as a specific diagnostic category. However, in many studies, DDD was grouped together with other forms of MPGN, which makes it difficult to make specific statements about DDD. A recent KDIGO (Kidney Disease: Improving Global Outcomes) controversies conference ^23^ recommended that all patients receive optimal blood pressure control and that patients with moderate disease—defined as urine protein of more than 500 mg/24 hours despite supportive therapy or moderate inflammation on renal biopsy or recent rise in creatinine—receive prednisone or mycophenolate mofetil (MMF). A recent case series suggested that MMF is effective in C3GN ^54^. In severe disease with proteinuria of more than 2 g/24 hours or in severe disease on biopsy or progressive creatinine increase, the KDIGO conference suggested the use of methylprednisolone pulse dosing as well as other anti-cellular immune suppressants. In many cases of C3 glomerulopathy, the deposition of C3 in glomeruli leads to subsequent activation of C5 and there is considerable interest in the possibility of using the anti-C5 antibody eculizumab for treatment. There are a number of case reports of use of eculizumab and one small open-label, non-blinded clinical trial of eculizumab for C3 glomerulopathy ^55^. Some of the patients have shown clinical improvement and in some there was reduction of glomerular inflammation on re-biopsy. However, the exact role of eculizumab remains to be defined, and the KDIGO conference concluded ‘Data are insufficient to recommend eculizumab as a first-line agent for the treatment of rapidly progressive disease’ ^23^. Rational treatment of C3 glomerulopathy would involve inhibition of C3 activation and there are a number of drugs that are in pre-clinical development that may achieve that and allow a more targeted therapy in the future.

## Outstanding questions

The recognition of C3 glomerulopathy has allowed us to recognise a group of patients in whom the disease mechanism is a defect in the control of the alternative pathway of complement. This has facilitated understanding of the role of genetic and acquired factors in driving disease. However, there are a number of problems still to be solved. Perhaps the most obvious is the fact that we do not understand why the kidney, and specifically the glomerulus, is targeted by complement in this disease. It is likely to be a combination of the physico-chemical properties of the glomerular basement membrane and the unique hemodynamics of the glomerulus. In terms of the pathological features, the reason why the deposits sometimes develop the features of DDD is still unknown. As noted above, the distinction between immune complex disease and C3 glomerulopathy is not always clear-cut and requires a better understanding of the natural history of the disease and the probable role of immune complexes in initiation and exacerbation. Finally, there is a need to better understand how the morphological features on biopsies relate to clinical presentation, underlying genetic and acquired factors and clinical outcome so that patients can be appropriately stratified in clinical trials.
